# Characterisation of breast cancer molecular signature and treatment assessment with vibrational spectroscopy and chemometric approach

**DOI:** 10.1371/journal.pone.0264347

**Published:** 2022-03-09

**Authors:** Magdalena Kołodziej, Ewa Kaznowska, Sylwia Paszek, Józef Cebulski, Edyta Barnaś, Marian Cholewa, Jitraporn Vongsvivut, Izabela Zawlik

**Affiliations:** 1 Medical College of Rzeszow University, Rzeszow, Poland; 2 Centre for Innovative Research in Medical and Natural Sciences, Medical College of Rzeszow University, Rzeszow, Poland; 3 Department of Pathology, Medical College of Rzeszow University, Rzeszow, Poland; 4 Department of Genetics, Institution of Experimental and Clinical Medicine, University of Rzeszow, Poland; 5 Centre for Innovation and Transfer of Natural Sciences and Engineering Knowledge, University of Rzeszow, Rzeszow, Poland; 6 Institute of Obstetrics and Emergency Medicine, Medical College of Rzeszow University, Rzeszow, Poland; 7 Synchrotron, ANSTO, Victoria, Australia; Arizona State University, UNITED STATES

## Abstract

Triple negative breast cancer (TNBC) is regarded as the most aggressive breast cancer subtype with poor overall survival and lack of targeted therapies, resulting in many patients with recurrent. The insight into the detailed biochemical composition of TNBC would help develop dedicated treatments. Thus, in this study Fourier Transform Infrared microspectroscopy combined with chemometrics and absorbance ratios investigation was employed to compare healthy controls with TNBC tissue before and after chemotherapy within the same patient. The primary spectral differences between control and cancer tissues were found in proteins, polysaccharides, and nucleic acids. Amide I/Amide II ratio decrease before and increase after chemotherapy, whereas DNA, RNA, and glycogen contents increase before and decrease after the treatment. The chemometric results revealed discriminatory features reflecting a clinical response scheme and proved the chemotherapy efficacy assessment with infrared spectroscopy is possible.

## Introduction

Triple-negative breast cancer (TNBC) is the most aggressive epithelial breast tumor, diagnosed in approximately 10–20% of all breast cancer patients [1]. TNBC is immunohistochemically negative for the protein expression of the estrogen receptor (ER) and progesterone receptor (PR), and lack of overexpression/gene amplification of hormone epi-dermal growth factor receptor 2 (HER2) is observed [2]. Approximately 70% of triple-negative breast cancer patients fail to achieve a pathologic complete response after chemotherapy due to the lack of targeted therapies for this subtype [3]. Besides, TNBC is associated with a significantly worse overall survival, and compared to the Luminal A subtype, the risk of death, recurrence, or metastasis is several times higher [4].

TNBC has been investigated with different diagnostic approaches, including physical and optical techniques [5–10]. Many of these require stains and labels to enhance contrast and thus can interfere with the actions of small metabolites and drugs. Besides, many of the available methods are time-consuming and incommodious. In contrast, vibrational spectroscopy, including Fourier transform infrared (FTIR) absorption, allows for detailed characterization of biological materials without using complicated sample preparation procedures or additional reagents [11, 12]. FTIR spectroscopy has been extensively used for different medical applications such as cancer research [13–15], stem cells [16], inflammatory diseases [17, 18], and more. The purpose of the current study was to monitor chemo-therapy in four female patients with the diagnostic approach developed based on the focal plane array (FPA) FTIR microspectroscopy and chemometric techniques to find spectral markers of treatment effectiveness.

## Material and methods

### Material

The study was conducted under the Institutional Review Board (Protocol No. KBET/6/06/2014) from June 2014 at the University of Rzeszow. All used in this study experimental protocols were approved by the Institutional Ethics Committees of the University of Rzeszow and were carried out following the approved guidelines. Informed consents were obtained from all subjects. The study was conducted based on formalin-fixed paraffin embedded (FFPE) breast tissue samples obtained through core biopsy from two healthy controls (breast reduction procedures) and four TNBC female patients before and after preoperative chemotherapy with different stages of malignancy. Characteristics of all patients are presented in S1 Table. FFPE breast cancer tissue sections of only tumor mass were microtomed into 5 μm thick sections and fixed on CaF2 substrates (Crystran, UK.). Our previous works proved that the material preparation methodology is suitable for FTIR spectroscopy and paraffin fixation did not alter chemometrics results [13, 19]

### Methods

**Fig 1:**
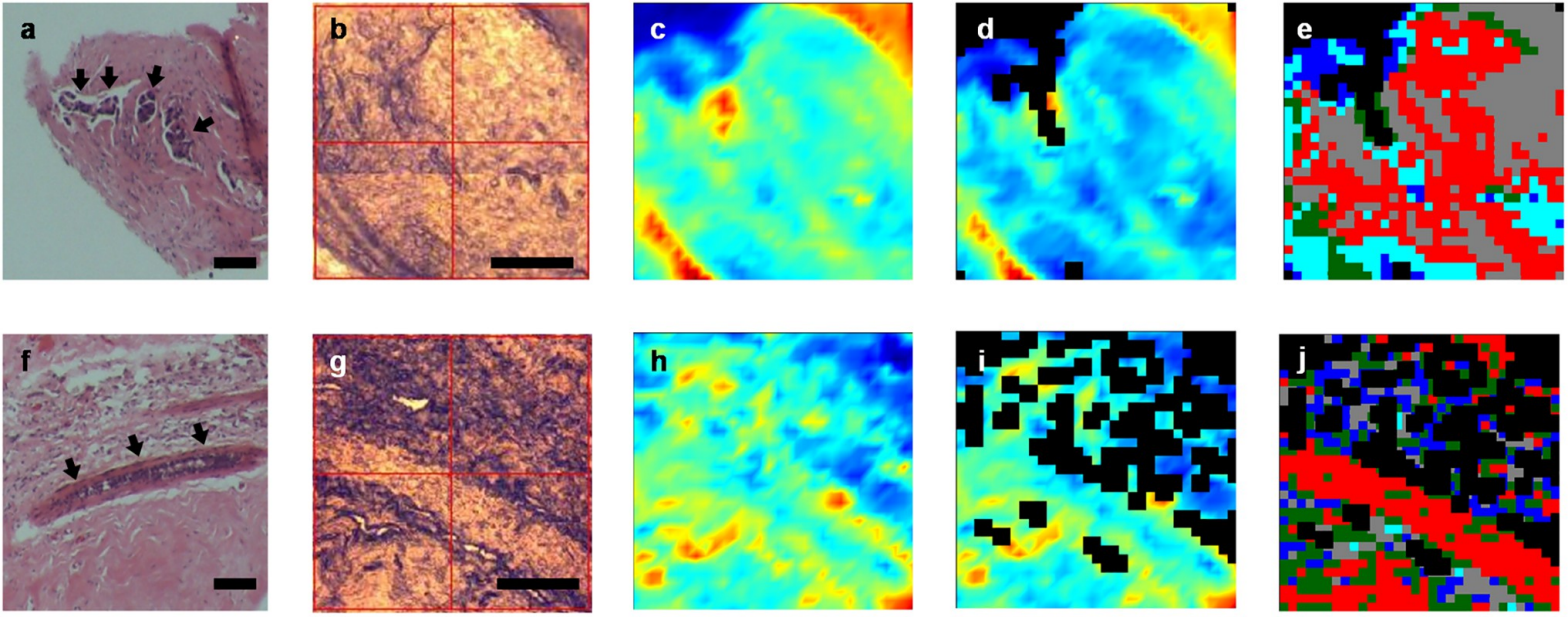
Schematic illustration of spectral pre-processing of tissue before (top) and after (bottom) chemotherapy. Presented: (a,f) microscopic images of H&E stained sections, scale bars: 100 μm; (b,g) OPUS images, scale bars: 100 μm; (c,h) FTIR chemical images of protein distribution using integrated area under amide I band (1710–1600 cm^−1^); (d,i) denoised and quality-tested FTIR chemical images; (e,j) corresponding hierarchical cluster analysis images. Spectra for further analysis were chosen based on acquired HCA maps–cluster corresponding to selected ROI were included into final PCA set.

Experiment was performed at the IRM beamline in Australian Synchrotron. As reported previously [13], spectra were collected in transmission mode within 4000-800cm^-1^ spectral region using a Bruker Hyperion 2000 FTIR microscope equipped with a liquid-N2 cooled 64 × 64 element FPA detector and 15× objective lens, coupled to a Vertex 70/70v FTIR spectrometer. Each spectral image encompasses a 32 × 32 array of spectra resulting from binning the signal from each square of 4 detectors and a single spectrum in each FTIR image stands for molecular information acquired from 10,6 μm × 10,6 μm area of the sample. Such approach enables fast scanning of large areas, which is more suitable for future clinical applications. Spectral images were collected with 4 cm^-1^ spectral resolution with 64 co-added scans, Blackman-Harris 3-Term apodization, Power-Spectrum phase correction, and a zero-filling factor of 2 using OPUS 7.2 imaging software (Bruker). The areas on the breast tissue samples were selected based on their corresponding H&E stained sections, targeting the areas of cancer nests. The spectral selection was based on pre-processed chemical image and only spectra that corresponded to cancer tissue were selected for further analysis. Schematic presentation of spectral selection is presented on Fig 1.

FPA-FTIR images were analyzed using Cytospec v. 1.4.02 (Cytospec Inc., Boston, MA, USA). Spectra embedded in each image were first denoised using the PCA approach (10 PCs), and quality screened to keep only high-quality spectra with a minimum S/N ratio of 100. Selected spectra were subsequently converted into the second derivative using the Savitzky-Golay algorithm with 3 polynomial order and 13 smoothing points.

### Data analysis

Spectral peaks were selected based on the second derivative spectra. For more objective, non-bias investigation, raw spectra were first normalized, baseline corrected, and averaged (OPUS Software). Subsequently, the resultant spectra were transformed into a second derivative (Savitzky-Golay algorithm, The Unscrambler 10.3 software, CAMO Software AS., Oslo, Norway), and all minima (wavenumbers) were precisely identified. To exclude the contribution of paraffin, only 1700-1495cm^-1^ and 1350–950 cm^-1^ regions were used in the final analysis. In an attempt to estimate absorbance ratios for each experimental group, the curve fitting was performed in the spectral regions 1700–1495 cm^-1^ and 1350–950 cm^-1^, and the absorbance values of selected underlying bands were determined. Additionally, the sum of bands assigned to amide I (1700–1600 cm^-1^, AI), amide II (1600–1500 cm^-1^, AII), and amide III-nucleic acids (1350–950 cm^-1^, LWN) regions were determined. Subsequently, the following absorbance ratios were calculated: AI/AII, Ph1/LWN, Ph2/LWN, RNA/LWN, GLYCO/LWN, and DNA/LWN. Selected ratio values were analyzed using Statistica 13.0 (TIBCO Software Inc 2017). Principal component analysis (PCA) was performed using The Unscrambler^®^ 10.5 software package (CAMO Software AS., Oslo, Norway). Extended Multiplicative Signal Corrected (EMSC) second derivative spectra were combined into one set to investigate similarities and differences of the healthy breast tissues and tissues before and after the course of chemotherapy. Subsequently, PCA with 7 PCs, using the NIPALS algorithm, was performed separately for each degree of malignancy.

## Results

### Spectral description

**Fig 2:**
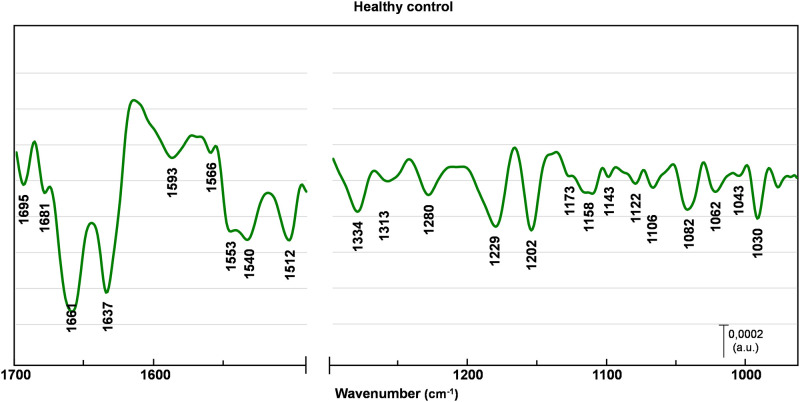
Healthy control averaged spectrum. EMSC-corrected 2nd derivative spectra of healthy control breast tissue with assigned minima.

**Fig 3:**
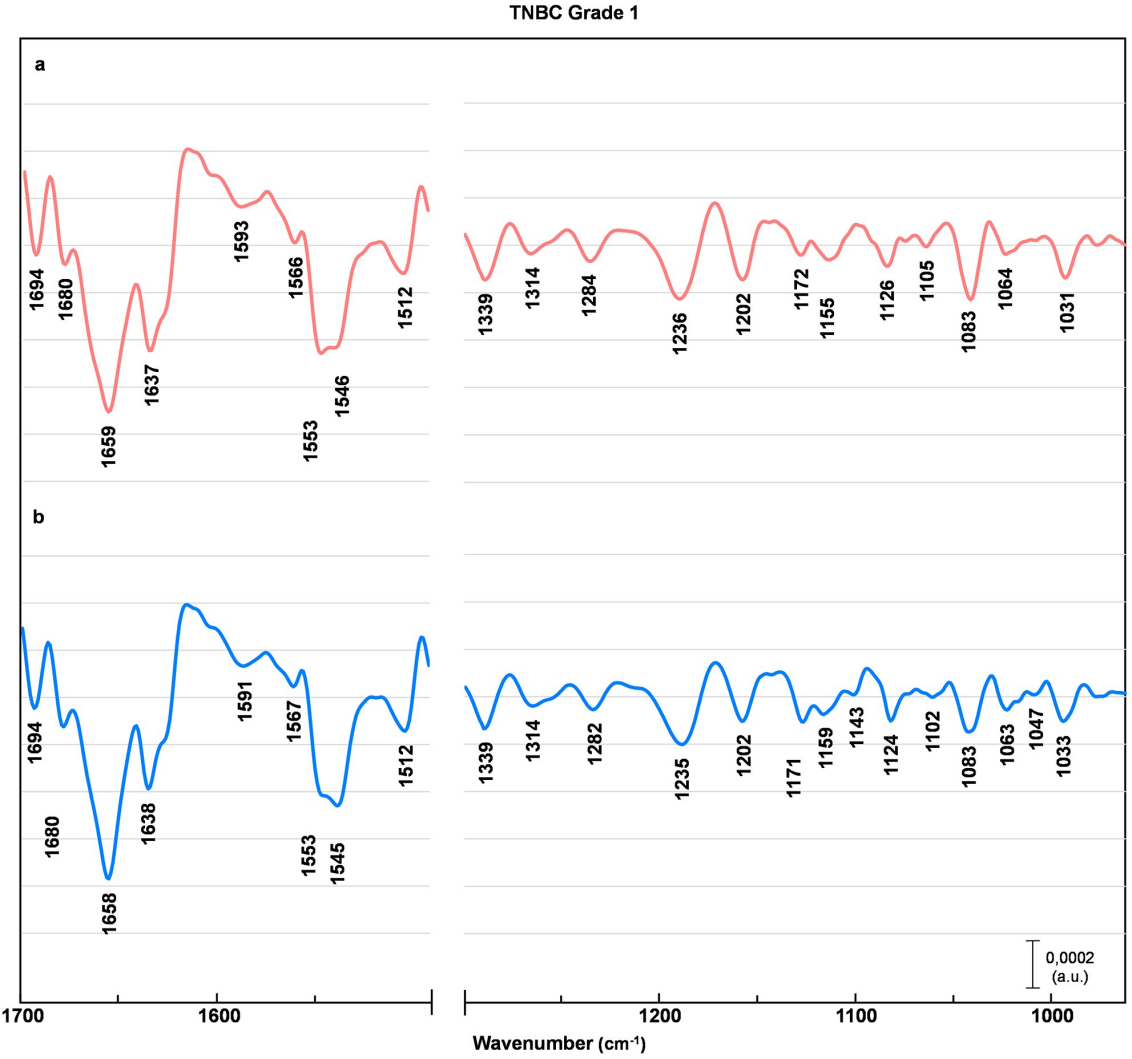
TNBC grade 1 averaged spectra. EMSC-corrected 2nd derivative spectra of TNBC grade 1 breast tissue before (a) and after (b) preoperative chemotherapy with assigned minima.

**Fig 4:**
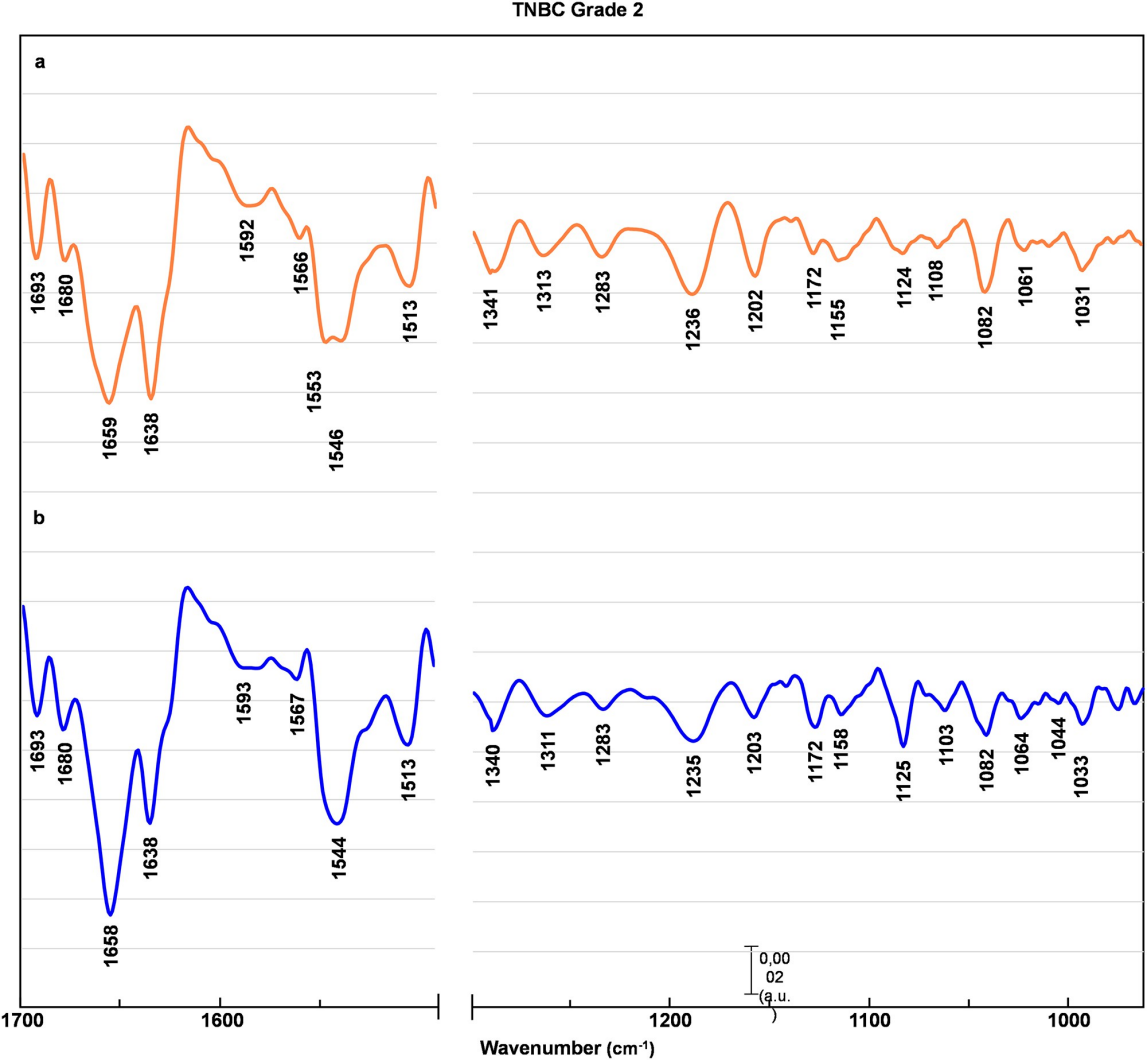
TNBC grade 2 averaged spectra. EMSC-corrected 2nd derivative spectra of TNBC grade 2 breast tissue before (a) and after (b) preoperative chemotherapy with assigned minima.

**Fig 5:**
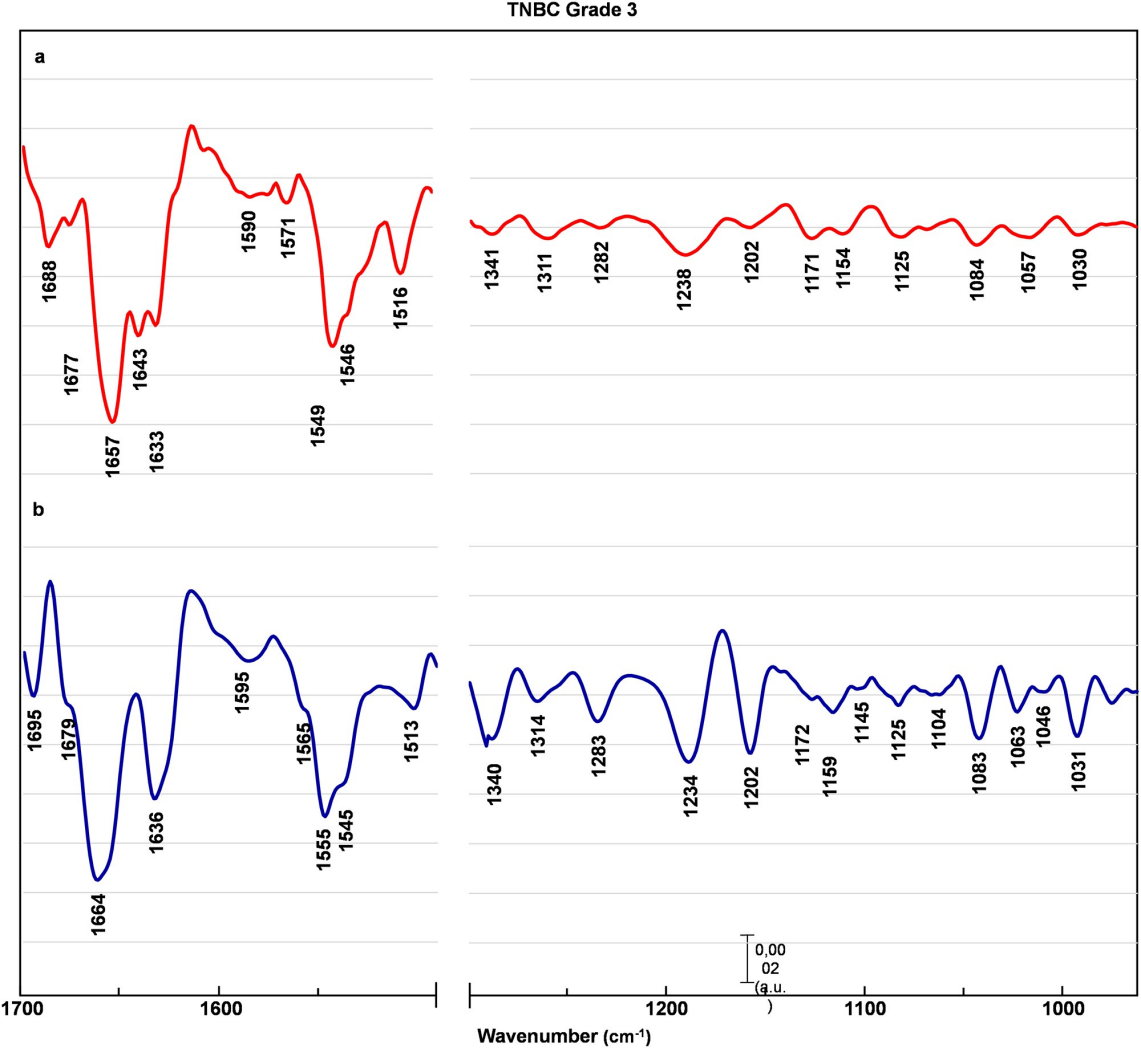
TNBC grade 3 averaged spectra. EMSC-corrected 2nd derivative spectra of TNBC grade 3 breast tissue before (a) and after (b) preoperative chemotherapy with assigned minima.

Figs 2–5 represents EMSC-corrected, second derivative averaged spectra obtained from healthy breast tissue (Fig 2), and tissue before (a) and after (b) chemotherapy (Figs 3–5). To examine the peaks positions the second derivative sets were used. All collected spectra are typically composed of peaks attributed to proteins, lipids, and nucleic acids. The minima observed within a region 1690–1630 cm^-1^ are assigned to α-helix (1661 cm^-1^), β-sheet (1695, 1637 cm^-1^) and β- turn (1681 cm^-1^) structures of amide I, with the majority of amide I proteins formed in α-helix structure [13, 14, 16, 20]. The most pronounced contrast between healthy control and before versus after chemotherapy was noted in the tissue with a G3 degree of malignancy (Fig 5). In G3 tumor stage before chemotherapy, the peaks located in amide I region (1681 cm^-1^, 1661 cm^-1^, 1637 cm^-1^) are shifted towards lower wavenumber by 4 cm^-1^, with the most pronounced change of the peak assigned to aggregated β-sheet, shifted by 7 cm^-1^. In the G3 after chemotherapy, these peaks return to the position similar to healthy control (Fig 5b). Of interest is a minimum also attributed to β-sheet conformation (1643 cm^-1^), found only in the G3 tumor stage before chemotherapy (Fig 5a). Less noticeable lesions were noted in G2 (Fig 4) and G1 (Fig 3) cancer stage.

A similar pattern have been observed in the amide II region (1590–1510 cm^-1^) [21–25]. In the case of G3 tumor stage before chemotherapy the peaks arise from C − N stretching coupled to N − H bending vibrations of amide II (1566 cm^-1^) [21] and C = C stretching vibrations of tyrosine (1512 cm^-1^) [24, 25] are shifted towards higher wavenumber by 5cm^-1^ and 4 cm^-1^ respectively (Fig 5a). Of interest is the minimum attributed to perpendicular modes of α-helix and parallel-chain β-sheet from amide II, found at 1540 cm^-1^ [23]. This band is shifted towards higher wavenumber by 6 cm^-1^ for all tumor stages before chemotherapy (Figs 3–5a), whereas after chemotherapy observed shifts are less pronounced (Figs 3–5b).

The examination of a lower wavenumber region reveal more pronounced differences not only in G3, but also in G1 and G2 cancer stage, with the most noticeable changes in minima arise from DNA, RNA and glycogen. Of note is also a minimum assigned to wagging vibrations of side chain in collagen (1334 cm^-1^) [15], shifted towards higher wavenumber by 5 cm^-1^ for G1 (Fig 3), 7 cm^-1^ before and 6 cm^-1^ after chemotherapy for G2 and G3 degree of malignancy (Figs 4 and 5). The peak assigned to asymmetric stretching of phosphodiester groups (1229 cm^-1^) [26] experienced the most significant changes. It is shifted towards higher wavenumber by 7 cm^-1^ before and 6 cm-1 after chemotherapy for G1 and G2 tumor stage (Figs 3 and 4), with even more pronounced shift of 9 cm^-1^ before and 5 cm^-1^ after treatment for G3 cancer stage (Fig 5). For the minimum assigned to (C − O) stretching vibrations from DNA (1062 cm^-1^) [20, 27] a shift by 5 cm^-1^ towards lower wavenumber were observed only in TNBC G3. The next affected peak is associated with ${PO}_{3}^{-2}$ asymmetric stretching from RNA (1122 cm^-1^) [15]. It is shifted towards higher wavenumber by 4 cm^-1^ for all tumor grades before chemotherapy, whereas there are no significant change in the spectra of tissues after chemotherapy in compare to healthy control. Similar changes occur for the peak assigned to (C − O) stretching vibrations of glycogen (1158 cm^-1^) [15] shifted towards lower wavenumber by 4 cm^-1^ in all tumor stages before chemotherapy, and returning to healthy control wavenumber values after treatment. Of note is another peak associated with glycogen (1043 cm^-1^) found only in healthy control and tissues after chemotherapy (Figs 2–5b). Also, the peak associated with oligosaccharides (1143 cm^-1^) can be found only in healthy control and after chemotherapy tissue in G1 and G3 tumor stage.

The summary of assigned wavenumbers, together with their biological origin, label and appropriate reference are reported in Table 1.

**Table 1:** Summary of mean values of wavenumbers (cm^−1^) seen in FTIR spectra of TNBC and control breast tissue. For each peak the vibrational mode and the label are reported.

Peak (cm^-1^)	Shifts						Band assignment	Label		Ref.
HC	G1		G2		G3					
	bf	af	bf	af	bf	af				
1695	1694	1694	1693	1693	1688	1995	Amide I: aggregated β-sheet structure	AI		[14, 16]
1681	1680	1680	1680	1680	1676	1679	Amide I: β-turn structure			[14, 16]
1661	1659	1658	1659	1658	1657	1664	Amide I: α-helix structure			[13, 20]
1637	1637	1637	1638	1638	1641	1636	Amide I: β-sheet structure			[14, 16]
1593	1593	1591	1592	1593	1590	1595	C = N N − H vibrations of adenine			[20]
1566	1566	1567	1566	1567	1571	1565	δas(N − H) and νs(C − N) vibrations of Amide II	AII		[21]
1553	1553	1553	1553	–	1549	1555	ν(C − O) and δ(N − H) vibrations of Amide II			[22]
1540	1546	1545	1546	1544	1546	1545	Amide II: δ(N − H) coupled to ν(C − N) vibrational mode			[23]
							Amide II: perpendicular modes of α-helix and parallel-chain β-sheet			
1512	1512	1512	1513	1513	1516	1513	C = C stretching vibrations from tyrosine			[24, 25]
1334	1339	1339	1341	1340	1341	1340	ω(CH_2_) vibrations of side chain in collagen	L W N		[15]
1313	1314	1314	1313	1311	1311	1314	ω(CH_2_) vibration from glycine			[15]
1280	1284	1282	1283	1283	1282	1283	Amide III band components of proteins			[27]
1229	1236	1235	1236	1235	1238	1234	$\nu as({PO}_{2}^{-})$ from DNA		Ph1	[26]
1202	1202	1202	1202	1203	1202	1202	$\nu as({PO}_{2}^{-})$ from DNA			[20]
1173	1172	1171	1172	1172	1171	1172	ν(C − O) and δ(C − O) from C − OH group (glycogen)		GLYCO	[15]
1158	1155	1159	1155	1158	1154	1159	ν(C − O) from polysaccharides			[15]
1143	–	1143	–	–	–	1145	Phosphate & oligosaccharides			[20]
1122	1126	1124	1124	1125	1125	1125	$\nu as{(PO}_{3}^{-2})$ from RNA		RNA	[15]
1106	1105	1102	1108	1103	–	1108	ν(CO), ν(CC), ring (polysaccharides)			[20]
1082	1083	1083	1082	1082	1084	1083	$\nu s({PO}_{2}^{-})$ from DNA		Ph2	[22]
1062	1064	1063	1061	1064	1067	1061	νs(C − O) from DNA; one of the triad peaks of nucleic acids		DNA	[20, 27]
							(along with 1031 and 1081 cm^-1^)			
1043	–	1047	–	1044	–	1046	νs(CO − O − C) from polysaccharides		GLYCO	[22]
1030	1031	1033	1031	1033	1030	1031	C − OH deformation of nucleic acids; one of the triad peaks of nucleic acids		DNA	[20, 21]
							(along with 1060 and 1081 cm^-1^)			

Abbreviations: G1-G3 = TNBC grades 1–3; HC = healthy control; bf = before chemotherapy; af = after chemotherapy; νs = symmetric stretch; νas = asymmetric stretch; δ = in-plane deformation (bend); ω = wagging vibration.

### Absorbance ratios calculation

**Fig 6:**
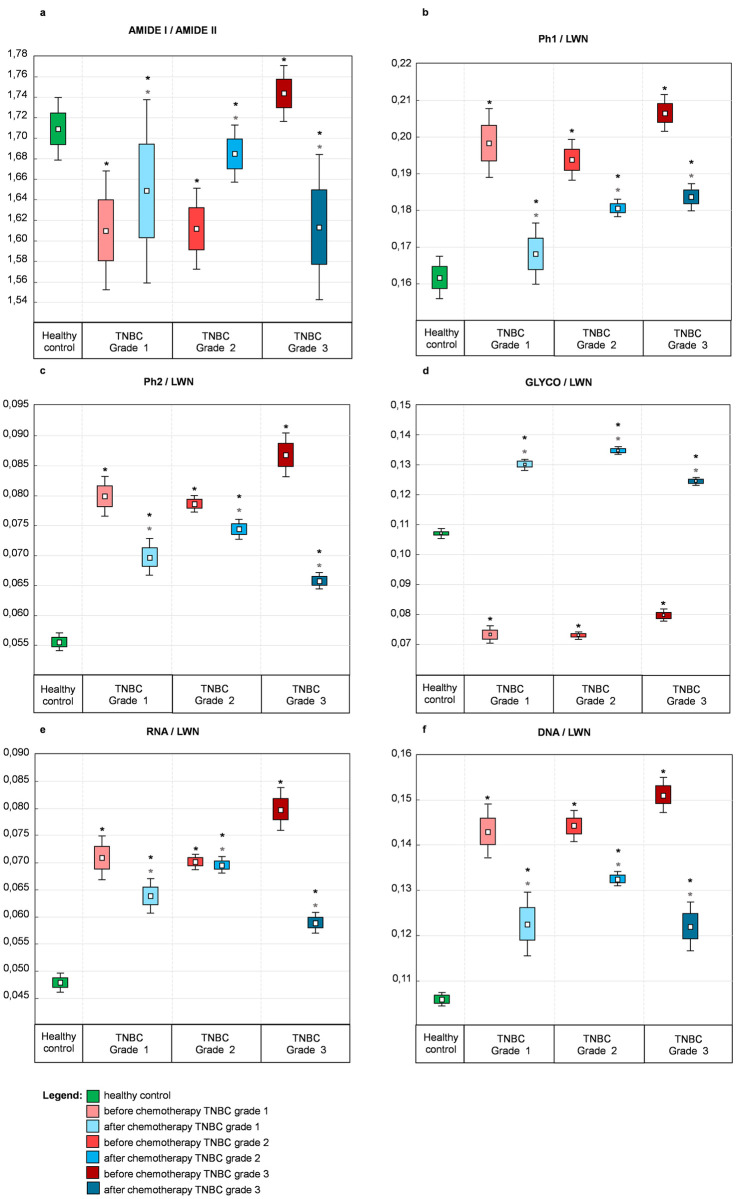
Box charts showing the numerical variation of the band area ratios. Ratios were calculated for healthy control and TNBC grades 1–3: (a) Amide I/Amide II, (b) Ph1/LWN, (c) Ph2/LWN, (d) GLYCO/LWN, (e) RNA/LWN, (f) DNA/LWN. The edges indicate standard deviation, the whiskers indicate standard deviation+1,96*standard deviation and the white square the mean. Stars over box charts indicate statistically significant difference between healthy control and TNBC patients (top, black) and before versus after chemotherapy (bottom, grey) determined with Wilcoxon test). Statistical significance was set at p<0,05.

The statistical analysis of the absorbance area ratios for healthy control, before and after chemotherapy patients in G1-G3 tumor stages are presented on Fig 6.

#### Amide I / Amide II ratio (1700–1500 cm^-1^)

The amide I / amide II ratio, reflecting the assessment of protein secondary structure [28], is presented on Fig 6a. For patients with G1 and G2 cancer stages the ratio before and after chemotherapy significantly decreases (G1&G2BF = 1,62±0,005; G1&G2AF = 1,67±0,018), but in both cases the difference between ratios of healthy control and after treatment is less pronounced (HC = 1,71). For G3 tumor stage, the ratio increases before and decreases after chemotherapy (G3BF = 1,74; G3AF = 1,61). The above findings are associated with an additional minimum attributed to β-sheet conformation (1643 cm^-1^), found only in G3 before chemotherapy (Fig 5a).

#### Amide III and nucleic acids (1350–950 cm^-1^)

The following results for ratios unravelling the amide III and nucleic acids formations in compare to healthy control have been achieved: Ph1/LWN ratio (amount of phosphate groups in proteins [29]) significantly increase (Fig 6b); Ph2/LWN ratio (amount of phosphate groups in nucleic acids [30]) significantly increase (Fig 6c); RNA/LWN ratio (RNA amount [31, 32]) significantly increase (Fig 6e); DNA/LWN (DNA amount [33]) significantly increase (Fig 6f). All the above ratios show a similar pattern for all three TNBC degrees of malignancy: the values before and after chemotherapy significantly increase, however the values after chemotherapy are closer to healthy control group. Moreover, the differences between healthy control and TNBC become more extensive with each tumor stage.

Interestingly, GLYCO/LWN ratio values, indicating the amount of carbohydrates [30], significantly decrease before and significantly increase after the treatment (Fig 6d). Such massive difference is associated with the glycogen peak observed at 1043 cm^-1^, absent in the tissues’ spectra before chemotherapy in all cancer stages (Figs 3–5a).

All discussed ratios presented as mean ± SD are summarized in S2 Table.

### Principal component analysis (PCA)

**Fig 7:**
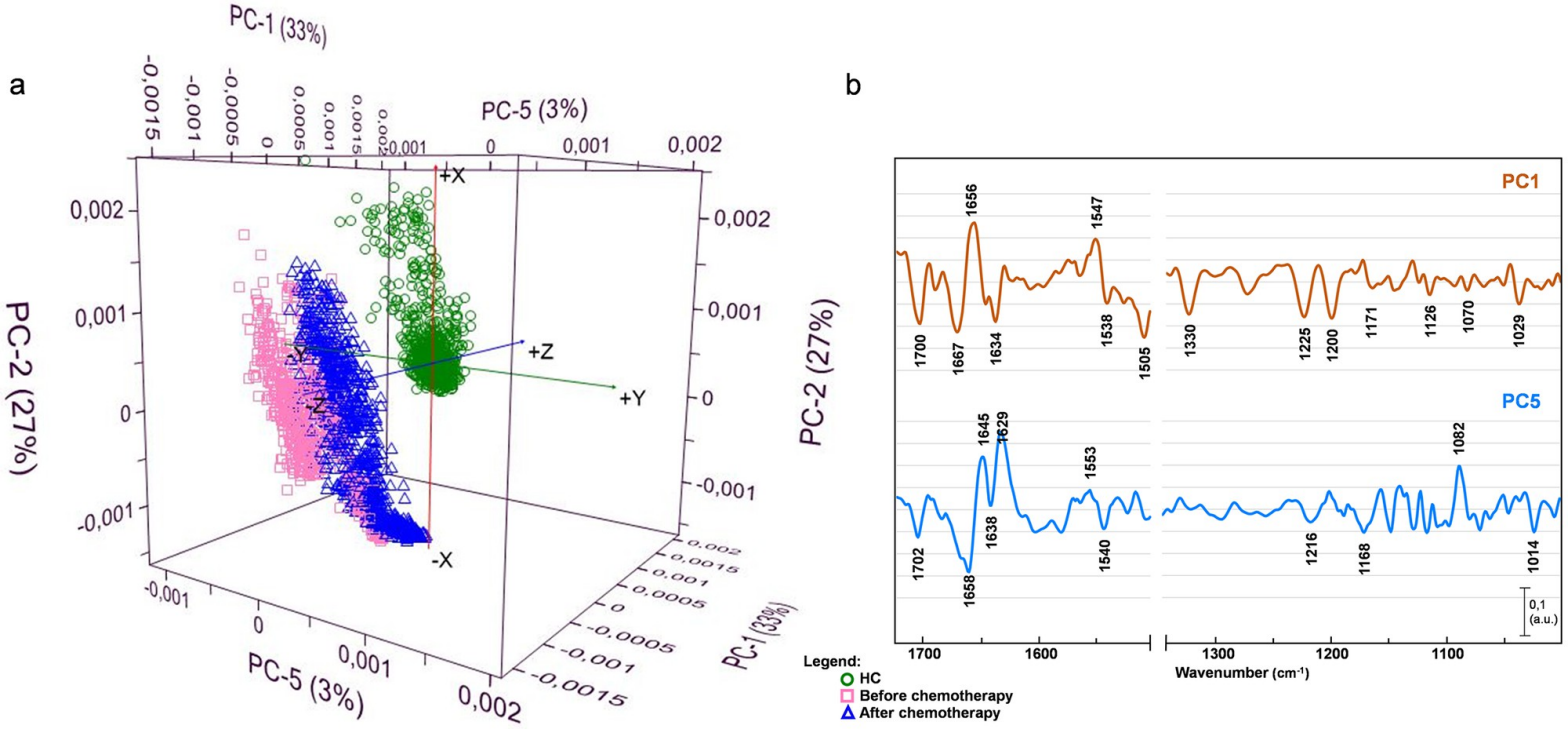
Principal component analysis performed on TNBC and healthy control spectral sets. PCA scores (a) and loadings (b) plots with the inclusion of the datasets from healthy control (green circle), before chemotherapy (pink square), and after chemotherapy (blue triangle).

The PCA analysis was performed using two spectral ranges: 1700–1495 cm^-1^ and 1350–950 cm^-1^, covering spectral features characteristic for proteins, lipids, carbohydrates, and nucleic acids. Initially, PCA was conducted on the entire spectral set (Fig 7) and subsequently repeated on separated spectral groups, divided by a TNBC degree of malignancy (S1 Fig).

The PC scores plot presents a distinct separation of healthy control and cancer tissue spectral clusters (Fig 7a). The PC loadings plot (Fig 7b) shows the amide I band region attributable to proteins (1700–1630 cm^-1^) [34] was heavily loaded for PC1, revealing separation of healthy control and malignant tissues with 33% explained variance. The negative PC1 loadings responsible for differentiation of healthy control from TNBC were found at 1700 cm^-1^ (C − O), 1667 cm^-1^ (amide I anti-parallel β-sheet), 1634 cm^-1^(amide I β-sheet), and 1538 cm^-1^(amide II). The positive PC1 loadings, explaining TNBC differentiation, were found at 1653 cm^-1^(amide I α-helix) and 1547 cm^-1^ (amide II perpendicular modes of α-helix and parallel-chain β-sheet).

In the lower wavenumber region the loadings responsible for distinction are located at 1330 cm^-1^ (collagen), 1225 cm^-1^ (DNA), 1200 cm^-1^ (DNA), 1171 cm^-1^ (glycogen), 1126 cm^-1^ (RNA), 1036 cm^-1^, and 1029 cm^-1^ (DNA) [35]. These findings appear to be in accordance with spectra (Figs 2–5) and ratios (Fig 6) examination, showing that proteins are most sensitive to mutation during carcinogenesis [25, 36], and nucleic acids play a substantial role in the process of tumor formation [37, 38].

In an attempt to separate spectral sets of before (BF) and after (AF) chemotherapy, we examined further PCs in the same PCA analysis. We did not notice distinct separation along PC2, PC3, and PC4 (see S2 Fig). However, going to further PCs, we found that PC5 (3% explained variance) shows a specified cluster pattern, with the loadings of the AF cluster separation similar to the loadings of healthy control distinction. In the higher wavenumber region (1720–1495 cm^-1^), negative loadings responsible for AF spectra separation can be found at 1702 cm^-1^ (C − O), 1658 cm^-1^ (amide I), 1638 cm^-1^ (amide I β-sheet), and 1540 cm^-1^ (amide II), whereas positive loadings arise from amide I (1645 cm^-1^; 1629 cm^-1^), and amide II (1553 cm^-1^) can explain BF spectral cluster distinction.

In the lower wavenumber region (1350–950 cm^-1^), the cluster of AF is distinguished by negative loadings arise from DNA (1216 cm^-1^; 1168 cm^-1^; 1014 cm^-1^), and the set of BF by strong positive loading attributed to symmetric stretching vibrations of the phosphate group from DNA (1082 cm^-1^).

## Discussion and conclusions

Vibrational spectroscopy techniques are increasingly applied for progression modeling in different cancer subtypes due to their ability to create label-free molecular fingerprint definition of crucial biological molecules. Spectral features of treatment effectiveness can be assessed concerning clinical responsiveness as well as in comparison to healthy control using both supervised and unsupervised analytical methods [39, 40]. Our previous studies reported a correlation between cancer tissue and FTIR spectral assessment [15, 41]. They proved the FTIR and multivariate data analysis approach is a suitable tool for detecting the changes of biochemical makeups that are the key to the treatment response. We also found that FPA-FTIR coupled to PCA can be helpful in the assessment of chemotherapy efficacy [13]. Nevertheless, comparison before-after chemotherapy within the same patient significantly reduces the inference for the general population. Indifference, our present study compared the combined sets of healthy control and patients before and after treatment. Additionally, we prepared a detailed spectral description and analyzed absorbance ratios defined previously to discuss aspects of impairment in ovarian endometriosis [42]. In our study amide I/ amide II ratio for G1 and G2 cancer stages shows a similar decrease before and increase after chemotherapy. The most affected seem to be G3 patient, showing protein secondary structure increase before and decrease after chemotherapy. The raw spectra examination revealed that the amide II protein region is emphasized before chemotherapy, but this imbalance disappears after treatment. These findings are also confirmed by PCA, which showed increased presence of amide I β-sheet conformations in the spectra before chemotherapy, stabilizing after the treatment in all three cancer grades. The relationship between the protein amount and carcinogenesis has been demonstrated by many researchers [43–47]. In cancer cells, protein functions are disturbed [44], and metabolic pathways impair proper cell growth [45, 46].

Observations of the lower wavenumber region also provide evidence for mutagenic aberrations [47–54]. It has been previously found that the differences in DNA and RNA oscillation frequency play a substantial role in healthy/breast cancer spectra discrimination [48]. These could be explained by a number of factors: (1) increased DNA content, possibly associated with necrosis and apoptosis of cancer cells [49]; (2) the presence of ${PO}_{2}^{-}$ stretching vibrations, possibly attributed to DNA damage caused by reactive oxygen species [50]; (3) accelerated metabolism of DNA/RNA in cancer cells, resulting in oscillatory deformations of the peak of C − H of adenine, higher in patients with cancer [51, 52]; (4) the presence of tumor-derived circulating DNA, found in blood plasma [53, 54].

In the presented study, the ratios of Ph2, RNA, GLYCO, and DNA are increased in the group before and decreased after, approaching the values of healthy control. The most interesting is GLYCO/LWN ratio, indicating the amount of carbohydrates [31]. It is significantly decreased before and increased after chemotherapy, and this massive difference is associated with the absence of one of the carbohydrates peak (1043 cm^-1^) in all cancer spectra before the treatment. These findings coincide with available knowledge about the so-called “Warburg effect” [55], explaining higher glucose metabolism noticed in cancer cells during the neoplastic process.

When discussing the lower wavenumber region, G2 patient treatment response needs to be further investigated. In the PCA scores plot (S1a and S1b Fig), the distinct separation of before and after clusters is visible. However, the loadings plot reveals an increased amount of DNA and RNA remain in spectra after chemotherapy. The examination of nucleic acid ratios shows almost no change in RNA (Fig 6e), and the least distinctive DNA ratio change (Fig 6d and 6f) for the spectral clusters after treatment. Luckily, the chemotherapy for this patient was successful, but the above findings indicate that the tissue of patient G2 after chemotherapy still possesses the most biochemical features of the malignant tissue than those investigated in this study G1 and G3.

Indeed, our research has several limitations, which surely decrease its robustness. Firstly, the long-term follow-up information about each patient would be invaluable to prove our findings. In this experiment, all patients are alive without recurrence or metastases, and thus we cannot present results for unsuccessful chemotherapy, which would be an invaluable insight into the usefulness of our approach. Considering the above, we cannot define the sensitivity and specificity of our method to determine a prognostic result. Secondly, the paraffin sample fixation might impact the results of the analysis. However, FFPE is a standard procedure for histopathology, and analyzed in this manuscript samples are scarce since they came from the same patient before and after chemotherapy. In an attempt to avoid fixation impact to spectral description and chemometric results, we excluded the paraffin bands.

Finally, the number of patients in our present experiment was too small to draw a definite conclusion. However, the availability of these samples is strongly limited due to the necessity of obtaining tissue twice from the same patient: before and after the full course of chemotherapy. Unfortunately, patients often die during chemotherapy or refuse to sign the consent for the second material collection. Therefore, a small number of samples precludes sophisticated statistical methods, together with test power evaluation; therefore, statistical inference is limited. The above conclusion suggests that it is essential to perform further studies with more samples to make the results significant for clinical practice. Still, together with previous results [13, 41], we demonstrated the treatment efficacy estimation is possible by examining the raw spectrum and applying different chemometric approaches alone. However, like other researchers, we suggest using different approaches combined to reveal various spectral aspects and obtain fundamental information about the disease’s nature.

## Supporting information

S1 Fig PCA results performed reflecting TNBC degree of malignancy. PCA scores (a, c, e) and loadings (b, d, f) plots showing projections against the first 3 PCs with the inclusion of datasets of healthy control (green) and G1 (a, b), G2 (c, d) and G3 (e, f) TNBC degree of malignancy.
(TIF)

S2 Fig PCA results of TNBC combined spectral set and healthy control. PCA scores showing projections against PC1/Pc2/PC3 (a) and PC1, PC2, PC4 (b).
(TIF)

S1 Table Clinicopathological characteristics of all patients. (DOCX)

S2 Table Absorbance ratios of healthy control and TNBC spectra. (DOCX)
