# Supplementary figures and images for: Characterisation of breast cancer molecular signature and treatment assessment with vibrational spectroscopy and chemometric approach
Source: PLoS One. 2022 Mar 9;17(3):e0264347. doi: 10.1371/journal.pone.0264347 (PMC8906614; doi:10.1371/journal.pone.0264347)

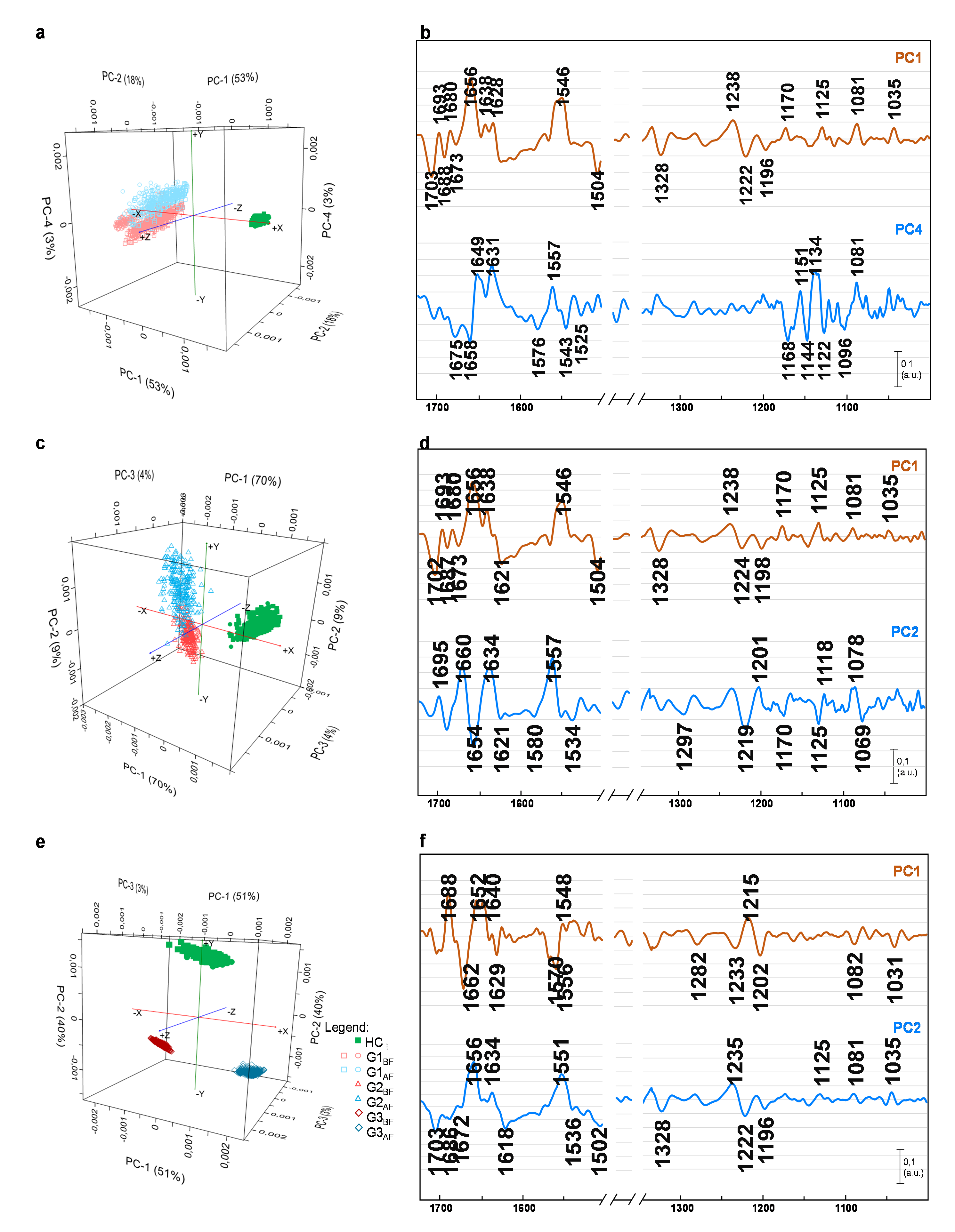

Supplement: S1 Fig — PCA scores (a, c, e) and loadings (b, d, f) plots showing projections against the first 3 PCs with the inclusion of datasets of healthy control (green) and G1 (a, b), G2 (c, d) and G3 (e, f) TNBC degree of malignancy. (TIF) [file pone.0264347.s001.tif]

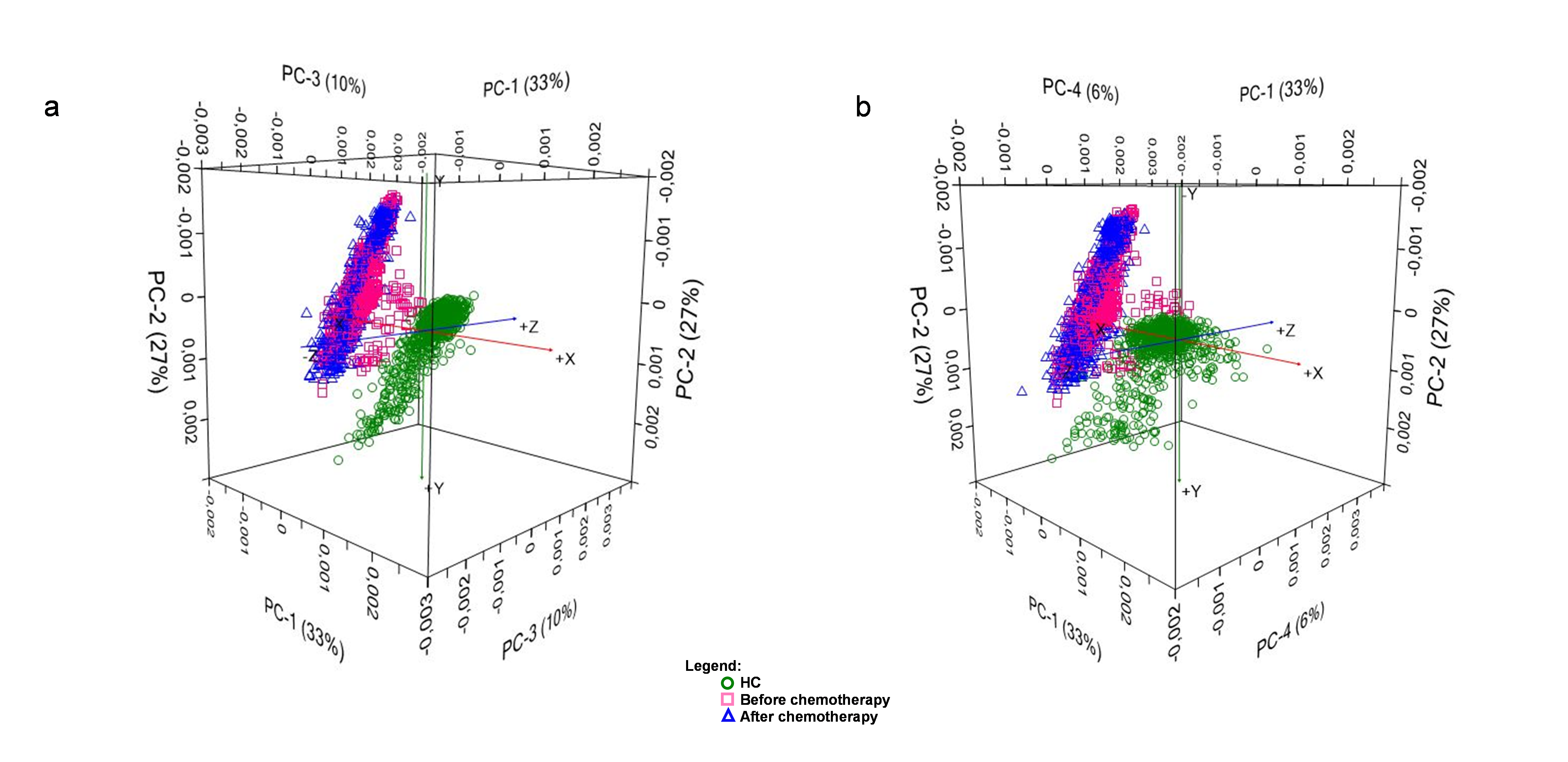

Supplement: S2 Fig — PCA scores showing projections against PC1/Pc2/PC3 (a) and PC1, PC2, PC4 (b). (TIF) [file pone.0264347.s002.tif]
