# Supplementary material for: Characterisation of breast cancer molecular signature and treatment assessment with vibrational spectroscopy and chemometric approach
Source: PLoS One. 2022 Mar 9;17(3):e0264347. doi: 10.1371/journal.pone.0264347 (PMC8906614; doi:10.1371/journal.pone.0264347)
Supplement: S1 Table — (DOCX) [file pone.0264347.s003.docx]

| Number | Grade | Age | Clinical staging before chemotherapy | Chemotherapy | Pathological response | Long term follow-up |
| --- | --- | --- | --- | --- | --- | --- |
| p1 | G3 | 36 | cT3N2 | AT6s - 5 series Adria  80 mg, Taxotere 150 mg | ypT1bNx | Alive without recurrence or  metastases |
| p2 | G1 | 44 | cT2N1 | AT 6 series Adria - 75 mg,  Taxotere – 115 mg | ypT2N3a | Alive without recurrence or  metastases |
| p3 | G1 | 59 | cT2N2 | FAC 6 series 5Fu-650mg,  Adria-65mg, CTX-650mg | ypT1aNx | Alive without recurrence or  metastases |
| p4 | G2 | 79 | cT2N2 | AT6s - 6 series Adria  90 mg, Taxotere 160 mg | ypT1aN1a | Alive without recurrence or  metastases |
