# Supplementary material for: Characterisation of breast cancer molecular signature and treatment assessment with vibrational spectroscopy and chemometric approach
Source: PLoS One. 2022 Mar 9;17(3):e0264347. doi: 10.1371/journal.pone.0264347 (PMC8906614; doi:10.1371/journal.pone.0264347)
Supplement: S2 Table — (DOCX) [file pone.0264347.s004.docx]

| Ratio | \| Mean \| \| --- \| | \| Minimum \| \| --- \| | \| Maximum \| \| --- \| | \| SD \| \| --- \| |
| --- | --- | --- | --- | --- | --- | --- | --- | --- |
| \| HC AI/AII \| \| --- \| | 1,709368 | 1,680678 | 1,762435 | 0,015541 |
| \| G1 bf AI/AII \| \| --- \| | 1,610455 | 1,539245 | 1,669914 | 0,029610 |
| \| G1 af AI/AII \| \| --- \| | 1,648796 | 1,552369 | 1,717784 | 0,045584 |
| \| G2 bf AI/AII \| \| --- \| | 1,611972 | 1,569605 | 1,659517 | 0,020261 |
| \| G2 af AI/AII \| \| --- \| | 1,685193 | 1,656059 | 1,721169 | 0,014232 |
| \| G3 bf AI/AII \| \| --- \| | 1,743690 | 1,703360 | 1,768093 | 0,013831 |
| \| G3 af AI/AII \| \| --- \| | 1,613565 | 1,442488 | 1,664961 | 0,036033 |
| \| HC Ph1/LWN \| \| --- \| | 0,161665 | 0,151292 | 0,165197 | 0,002957 |
| \| G1 bf Ph1/LWN \| \| --- \| | 0,198300 | 0,188559 | 0,208654 | 0,004765 |
| \| G1 af Ph1/TOT \| \| --- \| | 0,168153 | 0,157880 | 0,180263 | 0,004225 |
| \| G2 bf Ph1/TOT \| \| --- \| | 0,193820 | 0,190472 | 0,201434 | 0,002831 |
| \| G2 af Ph1/TOT \| \| --- \| | 0,180612 | 0,177940 | 0,184018 | 0,001128 |
| \| G3 bf Ph1/TOT \| \| --- \| | 0,206550 | 0,201752 | 0,216324 | 0,002570 |
| \| G3 af Ph1/TOT \| \| --- \| | 0,183693 | 0,179142 | 0,189393 | 0,001864 |
| \| HC Ph2/TOT \| \| --- \| | 0,055538 | 0,054369 | 0,057747 | 0,000735 |
| \| G1 bf Ph2/TOT \| \| --- \| | 0,079812 | 0,076433 | 0,085070 | 0,001692 |
| \| G1 af Ph2/TOT \| \| --- \| | 0,069715 | 0,066305 | 0,072672 | 0,001552 |
| \| G2 bf Ph2/TOT \| \| --- \| | 0,078591 | 0,077099 | 0,079951 | 0,000691 |
| \| G2 af Ph2/TOT \| \| --- \| | 0,074355 | 0,072261 | 0,075973 | 0,000828 |
| \| G3 bf Ph2/TOT \| \| --- \| | 0,086733 | 0,082174 | 0,089610 | 0,001858 |
| \| G3 af Ph2/TOT \| \| --- \| | 0,065740 | 0,064853 | 0,069234 | 0,000683 |
| \| HC RNA/TOT \| \| --- \| | 0,047873 | 0,046420 | 0,050423 | 0,000906 |
| \| G1 bf RNA/TOT \| \| --- \| | 0,070920 | 0,067997 | 0,078669 | 0,002071 |
| \| G1 af RNA/TOT \| \| --- \| | 0,063887 | 0,059702 | 0,066706 | 0,001609 |
| \| G2 bf RNA/TOT \| \| --- \| | 0,070196 | 0,068990 | 0,072099 | 0,000710 |
| \| G3 bf RNA/TOT \| \| --- \| | 0,079840 | 0,075937 | 0,082664 | 0,001930 |
| \| G3 af RNA/TOT \| \| --- \| | 0,058958 | 0,053186 | 0,060223 | 0,000986 |
| \| HC GLYCO/TOT \| \| --- \| | 0,107023 | 0,106290 | 0,108087 | 0,000336 |
| \| G1 bf GLYCO/TOT \| \| --- \| | 0,073259 | 0,070489 | 0,076930 | 0,001487 |
| \| G1 af GLYCO/TOT \| \| --- \| | 0,130010 | 0,128235 | 0,132582 | 0,000837 |
| \| G2 bf GLYCO/TOT \| \| --- \| | 0,073045 | 0,072225 | 0,074029 | 0,000380 |
| \| G2 af GLYCO/TOT \| \| --- \| | 0,134551 | 0,133639 | 0,136206 | 0,000619 |
| \| G3 bf GLYCO/TOT \| \| --- \| | 0,079705 | 0,077967 | 0,081721 | 0,000984 |
| \| G3 af GLYCO/TOT \| \| --- \| | 0,124425 | 0,123629 | 0,126472 | 0,000544 |
| \| HC DNA/TOT \| \| --- \| | 0,105902 | 0,104662 | 0,108673 | 0,000757 |
| \| G1 bf DNA/TOT \| \| --- \| | 0,143024 | 0,136802 | 0,147776 | 0,003011 |
| \| G1 af DNA/TOT \| \| --- \| | 0,122549 | 0,116516 | 0,131115 | 0,003619 |
| \| G2 bf DNA/TOT \| \| --- \| | 0,144228 | 0,140575 | 0,148315 | 0,001769 |
| \| G2 af DNA/TOT \| \| --- \| | 0,132466 | 0,130336 | 0,134223 | 0,000789 |
| \| G3 bf DNA/TOT \| \| --- \| | 0,151105 | 0,146248 | 0,158208 | 0,001943 |
| \| G3 af DNA/TOT \| \| --- \| | 0,121993 | 0,119093 | 0,137926 | 0,002743 |
